# Supplementary material for: Noninvasive investigation of the cardiodynamic response to 6MWT in people after stroke using impedance cardiography
Source: PLoS One. 2020 Jun 17;15(6):e0233000. doi: 10.1371/journal.pone.0233000 (PMC7299376; doi:10.1371/journal.pone.0233000)
Supplement: S1 Table — (DOCX) [file pone.0233000.s002.docx]

**S1 Table. Intraclass correlation coefficients of variables recorded at same time points over the two days.**

| Cardiodynamic parameters | Status | ICC_1,1_ | 95% CI | SEM | MDC_95% CI_ |
| --- | --- | --- | --- | --- | --- |
| HR(bpm) | At rest | 0.93 | 0.86-0.97 | 2.87 | 7.94 |
|  | 6WMT | 0.95 | 0.90-0.98 | 3.27 | 9.06 |
|  | recovery | 0.95 | 0.89-0.97 | 2.92 | 8.10 |
| SV(ml) | At rest | 0.92 | 0.84-0.96 | 4.44 | 12.32 |
|  | 6WMT | 0.92 | 0.83-0.96 | 5.31 | 14.71 |
|  | recovery | 0.87 | 0.74-0.94 | 5.97 | 16.55 |
| CO(l/min) | At rest | 0.90 | 0.81-0.95 | 0.35 | 0.96 |
|  | 6WMT | 0.93 | 0.85-0.97 | 0.58 | 1.60 |
|  | recovery | 0.90 | 0.80-0.95 | 0.43 | 1.19 |
| CI(l/min/m^2^) | At rest | 0.88 | 0.77-0.94 | 0.19 | 0.52 |
|  | 6WMT | 0.90 | 0.81-0.95 | 0.32 | 0.88 |
|  | recovery | 0.87 | 0.74-0.93 | 0.24 | 0.66 |
