# Supplementary material for: Noninvasive investigation of the cardiodynamic response to 6MWT in people after stroke using impedance cardiography
Source: PLoS One. 2020 Jun 17;15(6):e0233000. doi: 10.1371/journal.pone.0233000 (PMC7299376; doi:10.1371/journal.pone.0233000)
Supplement: S2 Table — (DOCX) [file pone.0233000.s003.docx]

**S2 Table. Time for each variable to reach a steady plateau and return to baseline (determined by the LSD method).**

| Cardiodynamic parameters | Rest | At the plateau | At the end of 6WMT | Time to plateau (min) | Returned to baseline (min) |
| --- | --- | --- | --- | --- | --- |
| HR(bpm) | 78±11 | 96±13 | 100±18 | 1.5 | 3.5 |
| SV(ml) | 71.3±16 | 88.1±19.6 | 89.3±18.6 | 0.5 | 3.5 |
| CO(l/min) | 5.5±1.2 | 8.4±2.0 | 8.9±2.6 | 1.5 | 3.5 |
| CI(l/min/m2) | 3±0.6 | 4.6±0.9 | 4.9±1.3 | 1.5 | 3.5 |
| SBP (mmHg) | 121.1±14.8 | NA | 131.6±16.7 | NA | 2.0 |
| DBP (mmHg) | 77.5±9.2 | NA | 80.9±10.3 | NA | 2.0 |

HR=heart rate; SV=stroke volume; CO=cardiac output; CI=cardiac index; SBP=systolic blood pressure; DBP=diastolic blood pressure. LSD=Least Significant Difference
