# Supplementary material for: Noninvasive investigation of the cardiodynamic response to 6MWT in people after stroke using impedance cardiography
Source: PLoS One. 2020 Jun 17;15(6):e0233000. doi: 10.1371/journal.pone.0233000 (PMC7299376; doi:10.1371/journal.pone.0233000)
Supplement: S3 Table — (DOCX) [file pone.0233000.s004.docx]

**S3 Table. Cardiodynamic responses before, during and after 6WMT.**

| Time | HR(bpm) | SV(ml) | CO(l/min) | CI(l/min/m^2^) | EF（%） |
| --- | --- | --- | --- | --- | --- |
| rest | 78±11 | 71.3±16.0 | 5.5±1.2 | 3.0±0.6 | 57.6±8.7 |
| 0.5 | 92±13 | 88.1±19.6 | 8.0±2.0 | 4.4±0.9 | 61.3±9.3 |
| 1 | 94±13 | 89.7±19.6 | 8.4±2.3 | 4.6±1.0 | 63.0±9.3 |
| 1.5 | 96±13 | 88.9±19.3 | 8.4±2.0 | 4.6±0.9 | 63.5±8.6 |
| 2 | 96±14 | 90.7±18.3 | 8.7±2.1 | 4.8±1.0 | 63.9±8.5 |
| 2.5 | 97±15 | 91.0±18.3 | 8.7±2.2 | 4.8±1.0 | 64.9±8.6 |
| 3 | 97±15 | 91.4±18.5 | 8.9±2.4 | 4.9±1.1 | 65.1±8.6 |
| 3.5 | 98±16 | 91.6±18.9 | 8.9±2.4 | 4.9±1.1 | 63.4±9.6 |
| 4 | 98±16 | 90.5±21.3 | 8.8±2.5 | 4.8±1.2 | 63.6±8.3 |
| 4.5 | 98±16 | 90.0±18.7 | 8.8±2.4 | 4.8±1.1 | 63.6±9.7 |
| 5 | 99±16 | 88.5±17.2 | 8.7±2.3 | 4.8±1.1 | 63.3±9.4 |
| 5.5 | 99±16 | 90.9±19.4 | 9.0±2.5 | 4.9±1.2 | 63.7±8.6 |
| 6 | 100±18 | 89.3±18.6 | 8.9±2.6 | 4.9±1.3 | 64.1±9.4 |
| post 0.5 | 89±14 | 80.0±17.5 | 7.1±1.9 | 3.9±0.9 | 62.0±9.1 |
| post 1 | 84±13 | 78.3±18.2 | 6.6±1.8 | 3.6±0.9 | 61.0±9.6 |
| post 1.5 | 82±14 | 76.9±17.0 | 6.3±1.8 | 3.5±0.9 | 59.9±9.3 |
| post 2 | 81±13 | 75.8±17.8 | 6.1±1.6 | 3.3±0.7 | 59.6±10.2 |
| post 2.5 | 80±13 | 75.3±18.2 | 6.0±1.5 | 3.3±0.7 | 58.7±9.2 |
| post 3 | 81±12 | 74.4±18.1 | 6.0±1.6 | 3.3±0.7 | 58.9±9.7 |
| post 3.5 | 80±13 | 72.9±15.3 | 5.8±1.4 | 3.2±0.7 | 58.0±10.0 |
| post 4 | 80±12 | 70.6±19.4 | 5.7±1.4 | 3.2±0.7 | 57.8±9.0 |
| post 4.5 | 80±13 | 72.7±17.6 | 5.7±1.5 | 3.2±0.7 | 60.9±18.6 |
| post 5 | 80±13 | 73.4±19.6 | 5.8±1.5 | 3.2±0.7 | 57.7±10.2 |
| post 5.5 | 80±11 | 72.7±17.6 | 5.7±1.4 | 3.1±0.7 | 58.1±9.4 |
| post 6 | 79±12 | 72.0±16.0 | 5.7±1.3 | 3.1±0.6 | 58.5±8.8 |
| post 6.5 | 79±12 | 71.7±16.9 | 5.6±1.3 | 3.1±0.6 | 57.5±8.6 |
| post 7 | 79±12 | 71.9±16.3 | 5.6±1.2 | 3.1±0.6 | 57.4±8.9 |
| post 7.5 | 80±12 | 70.7±17.7 | 5.5±1.4 | 3.1±0.6 | 57.2±9.2 |
| post 8 | 79±12 | 71.6±17.6 | 5.6±1.4 | 3.1±0.7 | 57.4±9.0 |
| post 8.5 | 79±12 | 70.8±15.7 | 5.5±1.2 | 3.0±0.6 | 57.7±8.8 |
| post 9 | 79±12 | 72.3±16.5 | 5.6±1.2 | 3.1±0.6 | 57.8±9.0 |
| post 9.5 | 79±12 | 70.9±16.1 | 5.5±1.2 | 3.0±0.6 | 57.4±8.6 |
| post 10 | 79±11 | 70.9±16.7 | 5.5±1.3 | 3.0±0.6 | 57.3±8.8 |
