# Supplementary material for: Noninvasive investigation of the cardiodynamic response to 6MWT in people after stroke using impedance cardiography
Source: PLoS One. 2020 Jun 17;15(6):e0233000. doi: 10.1371/journal.pone.0233000 (PMC7299376; doi:10.1371/journal.pone.0233000)
Supplement: S4 Table — (DOCX) [file pone.0233000.s005.docx]

**S4 Table. Correlation between 6MWD and cardiodynamic parameters at the end of 6WMT.**

| Cardiodynamic parameters | At the end of 6WMT | |
| --- | --- | --- |
|  | r_s_ | P value |
| HR | 0.46 | 0.013 |
| SV | 0.42 | 0.023 |
| CO | 0.66 | ＜0.001 |
| CI | 0.63 | ＜0.001 |

6MWD=6minute walk distance; 6MWT=6minute walk test; HR=heart rate; SV=stroke volume; CO=cardiac output; CI=Cardiac index
