# Supplementary material for: Noninvasive investigation of the cardiodynamic response to 6MWT in people after stroke using impedance cardiography
Source: PLoS One. 2020 Jun 17;15(6):e0233000. doi: 10.1371/journal.pone.0233000 (PMC7299376; doi:10.1371/journal.pone.0233000)
Supplement: S5 Table — (DOCX) [file pone.0233000.s006.docx]

| Time elapsed since diagnosis of stroke | | | | | | |
| --- | --- | --- | --- | --- | --- | --- |
|  |  | less than 1 year (n=19) | over 1 year (n=10) | Mean difference | P value | 95%CI |
| BMI  kg/m^2^ |  | 24.59±1.98 | 24.69±2.94 | 0.10 | P=0.91  ＞0.05 | -1.98 ~1.78 |
| BSA  (m^2^) |  | 1.76±0.14 | 1.91±0.15 | 0.15 | P=0.01  <0.05 | -0.27 ~-0.04 |
| Age (yr) |  | 56.84±11.04 | 53.10±10.67 | 3.74 | P=0.38  P＞0.05 | -5.01 ~12.49 |
| HR | At rest | 80.64 ±2.43 | 73.11±3.34 | 7.53 | P=0.08  ＞0.05 | -0.95~16.00 |
|  | At end of 6WMT | 103.26±4.02 | 93.39±5.55 | 9.87 | P=0.16  ＞0.05 | -4.18~23.93 |
| SV | At rest | 70.32±15.72 | 73.10±17.12 | 2.79 | P=0.66  ＞0.05 | -10.20~15.77 |
|  | At end of 6WMT | 87.05±18.26 | 93.44±19.63 | 6.39 | P=0.39  ＞0.05 | -8.62~21.41 |
| CO | At rest | 70.32±3.72 | 73.10±5.12 | 2.79 | P=0.66  ＞0.05 | -10.20~15.77 |
|  | At end of 6WMT | 87.05±4.30 | 93.44±5.92 | 6.39 | 0.39  ＞0.05 | -21.41~8.62 |
| CI | At rest | 3.16±0.58 | 2.79±0.45 | 0.36 | P=0.10  ＞0.05 | -0.07~0.80 |
|  | At end of 6WMT | 5.05±1.11 | 4.56±1.53 | 0.48 | P=0.34  ＞0.05 | -0.53~1.50 |

**S5 Table. Comparison of characteristics in subjects with time elapsed since stroke diagnosis longer or shorter than 1 year**

HR=heart rate, SV=stroke volume, CO=cardiac output, CI=cardiac index
