# Supplementary material for: Noninvasive investigation of the cardiodynamic response to 6MWT in people after stroke using impedance cardiography
Source: PLoS One. 2020 Jun 17;15(6):e0233000. doi: 10.1371/journal.pone.0233000 (PMC7299376; doi:10.1371/journal.pone.0233000)
Supplement: Attachment — Submitted filename: Respose to Reviewer 2-rev3.pdf [file pone.0233000.s011.pdf]

Responses to Reviewer 2's comments

Major Comments:

The manuscript is valuable in terms of methodology and results. Unfortunately, I have the impression that you are completely unable to explain whether the observed hemodynamic reaction is correct or not, whether it have been observed in other groups (not in PH!) already and what is clinical significance of your observations. I have no doubts about the 6MWT methodology. The intention of my questions 6 - 9 and 13 and 18 was a broader view on the obtained results in order to supplement and improve the discussion on their basis. Compare the obtained hemodynamic results to the results obtained in other, more pathophysiologically related diseases to stroke such as CAD or AH.

We are disappointed that Reviewer 2 has the misconception that we are 'completely unable' to explain whether the observed hemodynamic reaction is correct or not and 'whether it have been observed in other group (not in PH!)'.

We believe that Reviewer 2 appears to have failed to give appropriate weight to the primary aims of our study.

Our paper aims to report the cardiodynamic parameters recorded by Impedance Cardiography (ICG) **DURING a 6MWT** in a cohort of people after stroke. Our paper is the **first** to report SV, CO, CI **during** a 6MWT. The manuscript describes cardiodynamic parameters recorded by ICG during a 6MWT. There are limited publications from which to draw a reference and none for direct comparison, but our focus is cardiac parameters recorded DURING a 6MWT.

In a nutshell, our study describes the rationale for obtaining cardiodynamic data during a common clinical aerobic capacity test for people after stroke; explains how ICG was applied; reports the data recorded; and compares our data (recorded by ICG DURING a 6MWT) with reported literature on cardiodynamic variables obtained by ICG DURING a 6MWT in other patient cohorts.

The focus of our paper was on the potential role for ICG in the examination of cardiodynamic function in stroke patients. Tonelli et al. 2013 and Someya et al. 2015 were two excellent papers that describe clearly how to determine whether the main contributor to CO is SV or HR. Therefore, although these papers do not describe data from people with stroke, their work explains the analysis of cardiodynamic responses acquired by ICG during a 6MWT, highlighting the potential for ICG in further investigation during stroke rehabilitation; we believe their work is worthy of consideration.

In short, the focus of this article is **not** the cardiodynamics and aerobic capacity of people with stroke, but the measuring capability of ICG during a 6MWT in people with stroke.

Ad Q 9:

Do you know the entire manuscript? In this manuscript DOI 10.1080/10641963.2018.1523917:

Stronger correlations were observed between absolute value of VO2 and HR and VO2 and CO at peak exercise and changes in all of the evaluated parameters in subgroup of patient and reduced exercise capacity than with normal peak VO2 (>80% pred.)

I think this information is worth of consideration in the discussion.

Yes, we have considered and understand this article. This article described the cardiodynamic parameters obtained by ICG during a cardiopulmonary exercise test (CPET). The subjects also were subjected to a 6MWT. The correlation between the 6-minute walk distance (6MWD) achieved and the peak VO2, HR, SV and CO obtained during the CPET was investigated. This paper did NOT access cardiodynamic data DURING a 6MWT and therefore is irrelevant to the focus of our paper. This article forms the basis of the second phase of our study, which compares cardiodynamic data obtained by ICG during CPET and ICG data obtained during 6MWT.

Line 273-280

What can be the reason for the different contribution to CO by HR and SV observed by Comparing subgroups of patients: "time elapsed after stroke less than 1 year" and "time elapsed after stroke over 1 year"? Information on left ventricular dysfunction is insufficient in my opinion.

In our discussion we suggest that the lesser contribution of SV to CO might be associated with diastolic dysfunction. The contribution of SV to CO appeared to be higher in the patients in our cohort with the opportity for a longer period of rehabiliation training. This does nothing more than invite further investigation of the contributions of SV and HR to CO in response to exercise training. ICG is a convenient modality to determine this information. It is not the aim of our paper to discuss left ventricular dysfunction in our subject cohort.

Minor Comments:

Line 223-230 Good justification for the choice of method. It is recommended to move this fragment to the introduction as before. Please discuss the results in the discussion.

Line 242 Please complete the discussion with the sentence: The MRMI score reflects the mobility level of our cohort and correlates well with physical function variables such as range of movement and sensory.

Line 281 Please replace "but" with "and".

The above has been addressed accordingly.
