# Supplementary material for: Noninvasive investigation of the cardiodynamic response to 6MWT in people after stroke using impedance cardiography
Source: PLoS One. 2020 Jun 17;15(6):e0233000. doi: 10.1371/journal.pone.0233000 (PMC7299376; doi:10.1371/journal.pone.0233000)
Supplement: Attachment — Submitted filename: review.docx [file pone.0233000.s007.docx]

"Noninvasive investigation of the cardiodynamic response to 6MWT in people with stroke using impedance cardiography" is an interesting and valuable article. However, I have some questions and comments:

Line 16-17 "whereas HR, CO and CI continued to rise for 90 sec before plateau." Statement is unclear.

Line 130 Are you sure you mean CO not CI? You are writing about "body surface area ". Besides, heart rate was measured based on and R-R interval that derived from the electrocardiograph (ECG) not CO. Please correct.

Line 136 -141 Please do not duplicate information. Figure with arrangement of electrodes is enough, without description in main text. Do you have a one picture with the device placed on the patient with the electrodes?

Table 1 How many patients have been diagnosed with coronary artery disease, HFpEF and other serious cardiac diseases? Were there any differences in subgroups?

Line 176-177 I do not agree with "SV reached a steady plateau at 30 sec into the 6MWT, while HR, CO and CI reached a plateau after 90 sec". This is a non-physiological mechanism. An increase in HR and CO is observed until the end of 6MWT. Figure 3 and Figure 5 prove this. The increase in these parameters is not large, but seems continuous and gradual. Of course, I agree that the increase in CO is mainly the result of an increase in HR and not SV, and SV quickly reached plateau. This is a valuable observation.

Line 132 What is the value of haemodynamic parameters obtained in 1 second intervals during ICG? Is the average of value of parameters of the number of heartbeat in such an interval or the current value every 1 second? Please consider if beat-to-beat recorded data would be more reliable, especially when HR> 120 / min. Have additional ventricular or supraventricular beats been observed or have they occurred in past medical history? Did you observe artifacts while recording ICG data?

Table 2 How did you count the Ejection Fraction at rest and at the end of 6MWT? Was echocardiography performed? Please complete the description of the methodology.

Fig 4. Figure 4 seems to be useless, because you did not assess blood pressure during 6MWT or systemic vascular resistance. Values blood pressure at rest, at the end of the effort, and possibly the time to return it to baseline will be enough.

Fig5. We observe a big discrepancy between CO and CI. Did the subgroups differ significantly between BSA and BMI?

Line 243-244 Do you have any data on the difference in activity between subgroups? Was rehabilitation in the subgroup > 12 months after the stroke performed? Were they more fit, physically active?
