# Supplementary material for: Noninvasive investigation of the cardiodynamic response to 6MWT in people after stroke using impedance cardiography
Source: PLoS One. 2020 Jun 17;15(6):e0233000. doi: 10.1371/journal.pone.0233000 (PMC7299376; doi:10.1371/journal.pone.0233000)
Supplement: Attachment — Submitted filename: Responses_Reviewer2.docx [file pone.0233000.s008.docx]

**Reviewer# 2**

**We are grateful for Reviewer 2’s valuable comments, please see our responses to questions raised:**

1. Line 16-17 "whereas HR, CO and CI continued to rise for 90 sec before plateau." Statement is unclear.

***This sentence is now reworded. Please refer to page 2, line 16-18***

2. Line 130 Are you sure you mean CO not CI? You are writing about "body surface area ". Besides, heart rate was measured based on an R-R interval that derived from the electrocardiograph (ECG) not CO. Please correct.

***This sentence is now reworded. Please see page 8, line 131-136***

3. Line 136 -141 Please do not duplicate information. Figure with arrangement of electrodes is enough, without description in main text. Do you have one picture with the device placed on the patient with the electrodes?

***Text description is now deleted as suggested.***

4. Table 1 How many patients have been diagnosed with coronary artery disease, HFpEF and other serious cardiac diseases? Were there any differences in subgroups?

***The patient history was retrieved from hospital notes. Only 5 patients were diagnosed with coronary artery disease and there was no record of heart failure as a diagnosis. We are of the view that a subgroup analysis of such a small number would not be meaningful.***

5. Line 176-177 I do not agree with "SV reached a steady plateau at 30 sec into the 6MWT, while HR, CO and CI reached a plateau after 90 sec". This is a non-physiological mechanism. An increase in HR and CO is observed until the end of 6MWT. Figure 3 and Figure 5 prove this. The increase in these parameters is not large, but seems continuous and gradual. Of course, I agree that the increase in CO is mainly the result of an increase in HR and not SV, and SV quickly reached plateau. This is a valuable observation.

***The HR, SV and CO indeed continue to rise till the end of the 6MWT, however, the rise was much steeper in the first 90 sec for HR and the first 30 sec for SV; thereafter then the rise became more gradual (S3 Table, Fig 2). We have reworded the description of this phenomenon. Please refer to page 10, line 176-178.***

6. Line 132 What is the value of haemodynamic parameters obtained in 1 second intervals during ICG? Is the average of value of parameters of the number of heartbeat in such an interval or the current value every 1 second? Please consider if beat-to-beat recorded data would be more reliable, especially when HR> 120 / min. Have additional ventricular or supraventricular beats been observed or have they occurred in past medical history? Did you observe artifacts while recording ICG data?

***The Enduro^TM^ was used to capture cardiography signals. Signals were recorded at 1-second intervals. We did not observe any abnormal ventricular or supraventricular conduction abnormality.***

7. Table 2 How did you count the Ejection Fraction at rest and at the end of 6MWT? Was echocardiography performed? Please complete the description of the methodology.

***Ejection fraction was computed by the software. As this was not reported in other studies listed in Table 2, we have now deleted this variable from Table 2.***

8. Fig 4. Figure 4 seems to be useless, because you did not assess blood pressure during 6MWT or systemic vascular resistance. Values blood pressure at rest, at the end of the effort, and possibly the time to return it to baseline will be enough.

***Figure 4 is now deleted. Blood pressures before and immediately after 6MWT are displayed in S2 Table***

9. Fig5. We observe a big discrepancy between CO and CI. Did the subgroups differ significantly between BSA and BMI?

***There were no statistical differences in BMI between the two groups. However computed BSA was higher in the group with time elapsed after stroke-diagnosis longer than 12 months. This may explain the higher CI in this group, but the difference in CI did not reach statistical significance. We have now included an additional table (S5 Table) to illustrate the differences in characteristics between the groups.***

10. Line 243-244 Do you have any data on the difference in activity between subgroups? Was rehabilitation in the subgroup > 12 months after the stroke performed? Were they more fit, physically active?

***Unfortunately, we do not have detailed information on the activity of our patients. We do however know that the subjects with a longer time-elapsed after stroke diagnosis engaged in a longer duration of a walking program. We have included a more detailed discussion of this point. Please refer to page 14, line 243-251.***
