# Supplementary material for: Noninvasive investigation of the cardiodynamic response to 6MWT in people after stroke using impedance cardiography
Source: PLoS One. 2020 Jun 17;15(6):e0233000. doi: 10.1371/journal.pone.0233000 (PMC7299376; doi:10.1371/journal.pone.0233000)
Supplement: Attachment — Submitted filename: Response to reviewers.docx [file pone.0233000.s009.docx]

**Responses to Reviewer 2’s Comments**

1. Line 45-47 The sentence: "The assessment... capacity." is not needed.

*This sentence was not intended to be read in isolation but together with the fact that 6MWT is commonly used for assessment of aerobic capacity in people with stroke. Please see page 4, line 45 to 47.*

2. Line 61 Are you sure that references 15-18 are about exercise ICG?

*References 15 to 18 are now replaced with a recent review article on clinical application of ICG [new reference 17]. Please kindly see page 5, line 61-63.*

3. Line 63 References 19-21 I think that more accurate study is for example: doi: 10.1007/s10554-019-01738-y than studies carried out in children.

*We have now deleted the references to paediatric patients and replaced them with more recent publications, including the one suggested by the reviewer [Reference 18].*

4. Line 110-111 What is the significance of constant temperature and humidity for 6 MWT, please provide references.

*It is well known that variable temperature and humidity may influence exercise performance. Our subject cohort performed the sub-maximal exercise test in an environment where humidity and temperature were controlled. These criteria are not required by the ATS Guideline and so in order not to cause confusion, we have now removed the information on the temperature and humidity.*

5. Line 165-166 and line 169-170 repeated sentence "The distance covered in the second 6MWT was slightly greater than the first 6MWT"

*The first sentence refers to the ‘mean’ distance recorded and later refers to individual performance. To avoid the confusion, we have removed the second sentence. Please refer to line 166-169.*

6. Line 190-191 Do you know other studies (on other populations) describing the correlation of 6MWT with SV, HR, CO? Describe in the discussion.

*We have now added a paragraph describing correlation of 6MWT with SV, HR, and CO and compared our data with those reported in other populations. Please refer to page 15, line 250-260.*

7. Table 2 Are there studies with the use of 6MWT in case of diseases that are etiopathologically close to stroke? Hemodynamic studies during 6MWT or CPET with CAD, with AH, lack of training (sedentary lifestyle)? I understand that the model study was Tonelli's study but the PH pathogenesis is radically different from stroke, unless we are interested in the control group. Please comment on absolute values and parameter changes, are they larger / smaller? What can affect the observed hemodynamic profile in patients after stroke?

*Table 2 illustrates four studies [31,30,28,5] reporting heart rate changes associated with 6MWT. However SV, CO and CI data were not available in these studies. Our study is the first to report the SV, CO and CI response to a 6MWT in people after stroke. We have now highlighted that the ‘change’ in heart rate refers to an ‘increase’ in heart rate. Please refer to page 14, line 232.*

8. Line 203-204 The absolute values between the subgroups did not differ significantly, is not it? How can you explain the different percentage of linear regression? Were different trends in parameter changes observed between the subgroups? Which trend of changes is correct? Is any parameter trend a compensatory response? What could be the reason for the different results?

*We presume the reviewer is referring to lines 204-213 in the previous version. Linear regression analysis was a method used by Tonelli and colleagues [Reference 25] to determine whether the primary contribution to CO was HR or SV. We considered it meaningful to assess whether people with a longer post-stroke duration responded differently to exercise stress in the form of 6MWT. We have therefore adopted the same statistical method used by Tonelli and colleagues*.

9. In the subgroup > 12 months after stroke, worse physical capacity was observed, but a greater relationship between CO and SV and HR. See DOI:

10.1080/10641963.2018.1523917

*The reference suggested by the reviewer compared exercise capacity assessed via a 6MWT and cardiopulmonary exercise test with haemodynamic assessment via ICG in patients with arterial hypertension. The study concluded that ICG is a reliable method for assessing cardiovascular response to exercise. We are unsure how this relates to physical capacity in our subjects >12months after stroke.*

10. Line 223 - 228 should be put in the introduction rather than in the discussion.

*We have now rewritten the first paragraph of our Discussion. Please refer to lines 221-228.*

11. According Ref. 39 "6MWT is not, by itself, an adequate measure of aerobic fitness early after stroke". Please explain the choice of 6MWT as a research method in a different way.

*Justification for the use of 6MWT in the assessment of cardiorespiratory fitness for people after stroke, is now included with an additional reference. Please see lines 221-228.*

12. Line 230 -237 do not repeat information: The mean distance covered by our cohort of patients was 255m;... comparable to 274 m reported by Pradon" oraz "...that Pradon’s cohort achieved similar distance to our cohort...". Please edit these sentences.

*We were confused as to whether the reviewer prefers us using the absolute values to illustrate our discussion point or a ‘similar’ distance statement was sufficient. The second sentence, which appears to reiterate that a similar distance was covered in both studies, is in fact necessary to highlight that ‘despite the similar distance’, the reported rate of perceived exertion was very different. Please refer to line 230-236.*

13. Line 186-188 and line 237-239 Does it mean that 6MWT is performed according to the maximum capabilities? What could affect this result?

*It is expected that a 6MWT is performed at the subject’s maximum capacity. While the VO_2peak_ achieved at the end of a 6MWT is very similar to that obtained from a cycle graded exercise test (for example current reference 35), a graded cardiopulmonary exercise test remains the gold standard for assessment of maximum capacity. We followed the ATS guideline and delivered standard instructions to our subjects to attain their maximum performance. The results (% MHR at the end of 6MWT) are comparable to data reported by others, so we do not anticipate a problem with this methodology. The aim of our study was to illustrate that ICG can be used to measure haemodynamic parameters during a 6MWT and easily applied in a clinical setting. Construct validity of 6MWT performance as a measure of functional walking capacity in people with acute, subacute and chronic stroke has been well established [Reference36], and is beyond the scope of this current study.*

14. Line 239-242 What is the influence of 6MWT " with or without a walking stick, although some were unable to independently negotiate stair climbing". Please give the references. Was MRMI score result good or poor? Does it influence 6MWT, please give references.

*Our subjects were able to mobilize independently although a few required a walking cane. The number of those who required a walking cane is small and therefore subgroup analysis is not appropriate. ATS guidelines only require a record of whether the subject required a walking aid or not. Table 1 illustrates the high MRMI score in our patients. This is further explained in the discussion (please refer to page 14, line 240-241). The MRMI score reflects the mobility level of our cohort and correlates well with physical function variables such as range of movement and sensory function (doi:10.1589/jpts.28.2389). That being said, it is not a measurement tool for aerobic capacity. Detailed investigation of the relationship between MRMI and 6MWD is beyond the scope of our study.*

15. Line 250-256 Please at first discuss your results and then comment this on other research. Do not describe other authors's researches.

*We appreciate this may be true in most circumstances, however in our view, the concept would be more difficult for the readership to grasp if we commenced with data from a population of stroke survivors. We consider it is easier to first explain the scenario in a normal healthy population in various age groups*.

16. Line 260-266 "Tonelli.... [29]" What does this information bring in to discussion on the hemodynamic response in stroke patients? I understand that you methodologically modeled the study on Tonelli's work, however this can be entered in the methodology. What is the relationship between PH and stroke, how to explain the similarity of hemodynamics?

*We have now deleted the paragraph reiterating the linear regression method employed by Tonelli.*

17. Line 268 "While the changes in CO were similar". Were SV and HR changes also similar?

*Yes, all changes were similar. Please refer to supplementary table 5. ( S5 Table 5).*

18. Line 274-278 How do you explain the shorter 6MWTD after a year? Did they exercise more or less? Are you suggesting that stroke patients in the study group had LVDD? In reply to question 4 was: "there was no record of heart failure as a diagnosis". What is the connection to LVDD?

*The shorter 6MWD shown in Table 3 does not refer to a shorter 6MWD ‘after a year’. Please refer to Table 1, which shows that the duration of post stroke time in our subjects ranged from 1 month to 60 months. These sentences only postulate that diastolic function in patients after stroke may be influenced by exercise or activities. We did not at all suggest that our patients have left ventricular diastolic dysfunction. We have reworded the respective sentence to reflect our speculation. Please see page 16, line 281-282.*

19. Line 279- 280 Is this observation exceptional? See: doi: 10.1164/rccm.200203-166OC, doi: 10.1016/S0002-8703(03)00119-4,

*The learning effect with 6MWT is well documented in people with respiratory disease, however in people after stroke, reliability was shown to be excellent with one 6MWT trial and a single 6MWT is recommended in people after stroke (Reference 36). This is now highlighted in the Discussion, page 16, line 284-286.*

20. Line 288-289 What research was the basis for suspecting a relationship between the age and sex with hemodynamic parameters? Please provide references.

*We have now included two references [39,40] to support our view that age and gender influence cardiovascular function parameters. Please see page 17, line 295-296.*
