# Supplementary material for: Noninvasive investigation of the cardiodynamic response to 6MWT in people after stroke using impedance cardiography
Source: PLoS One. 2020 Jun 17;15(6):e0233000. doi: 10.1371/journal.pone.0233000 (PMC7299376; doi:10.1371/journal.pone.0233000)
Supplement: Attachment — Submitted filename: Review 3.docx [file pone.0233000.s010.docx]

**"Noninvasive investigation of the cardiodynamic response to 6MWT in people after stroke using impedance cardiography".**

**Major Comments:**

The manuscript is valuable in terms of methodology and results. Unfortunately, I have the impression that you are completely unable to explain whether the observed hemodynamic reaction is correct or not, whether it have been observed in other groups (not in PH!) already and what is clinical significance of your observations. I have no doubts about the 6MWT methodology. The intention of my questions 6 - 9 and 13 and 18 was a broader view on the obtained results in order to supplement and improve the discussion on their basis. Compare the obtained hemodynamic results to the results obtained in other, more pathophysiologically related diseases to stroke such as CAD or AH.

Ad Q 9:

Do you know the entire manuscript? In this manuscript DOI 10.1080/10641963.2018.1523917:

Stronger correlations were observed between absolute value of VO2 and HR and VO2 and CO at peak exercise and changes in all of the evaluated parameters in subgroup of patient and reduced exercise capacity than with normal peak VO2 (>80% pred.)

I think this information is worth of consideration in the discussion.

Line 273-280

What can be the reason for the different contribution to CO by HR and SV observed by Comparing subgroups of patients: "time elapsed after stroke less than 1 year" and "time elapsed after stroke over 1 year"? Information on left ventricular dysfunction is insufficient in my opinion.

**Minor Comments:**

Line 223-230 Good justification for the choice of method. It is recommended to move this fragment to the introduction as before. Please discuss the results in the discussion.

Line 242 Please complete the discussion with the sentence: The MRMI score reflects the mobility level of our cohort and correlates well with physical function variables such as range of movement and sensory.

Line 281 Please replace "but" with "and".
